# Validity of Amino Terminal pro-Brain Natiuretic Peptide in a Medically Complex Elderly Population

**DOI:** 10.4021/jocmr606w

**Published:** 2011-07-26

**Authors:** Mazhar A. Afaq, Azadeh Shoraki, Oleg Ivanov, Janardhan Srinivasan, Lawrence Bernstein, Stuart W. Zarich

**Affiliations:** a Departments of Medicine (Division of Cardiovascular Medicine) and Pathology, Bridgeport Hospital, Bridgeport, CT., Yale University School of Medicine, USA

## Abstract

**Background:**

The routine use of natiuretic peptides in severely dyspneic patients has recently been called into question. We hypothesized that the diagnostic utility of Amino Terminal pro Brain Natiuretic Peptide (NT-proBNP) is diminished in a complex elderly population.

**Methods:**

We studied 502 consecutive patients in whom NT-proBNP values were obtained to evaluate severe dyspnea in the emergency department. The diagnostic utility of NT-proBNP for the diagnosis of congestive heart failure (CHF) was assessed utilizing several published guidelines, as well as the manufacturer’s suggested age dependent cut-off points.

**Results:**

The area under the receiver operator curve (AUC) for NT-proBNP was 0.70. Using age-related cut points, the diagnostic accuracy of NT-proBNP for the diagnosis of CHF was below prior reports (70% vs. 83%). Age and estimated creatinine clearance correlated directly with NT-proBNP levels, while hematocrit correlated inversely. Both age > 50 years and to a lesser extent hematocrit < 30% affected the diagnostic accuracy of NT-proBNP, while renal function had no effect. In multivariate analysis, a prior history of CHF was the best predictor of current CHF, odds ratio (OR) = 45; CI: 23-88.

**Conclusions:**

The diagnostic accuracy of NT-proBNP for the evaluation of CHF appears less robust in an elderly population with a high prevalence of prior CHF. Age and hematocrit levels, may adversely affect the diagnostic accuracy off NT-proBNP.

**Keywords:**

Congestive Heart Failure; Natriuretic peptides; Diagnosis; Elderly Patients

## Introduction

Congestive heart failure (CHF) is a major cause of death and disability worldwide. In the United States alone there are an estimated 550,000 new cases of CHF yearly and it is projected that 10 million patients will have symptomatic heart failure within the next 30 years [1]. The economic cost of CHF is estimated at $56 billion per year, 70% of which is spent on hospital utilization.

Diagnosing CHF by clinical exam can be challenging in patients with multiple co-morbidities [2], but its accuracy is paramount both for patient care and the appropriate utilization of hospital resources. A careful history and clinical examination combined with additional laboratory data from currently available natriuretic peptide assays may provide the optimal balance of sensitivity and specificity [3] in the diagnosis of CHF. Natriuretic peptide testing has been FDA approved since 2002 and is now considered a Class IIa/Level of Evidence A recommendation for use in the evaluation of dyspnea in the emergency department according to current CHF guidelines [4].

Although natriuretic peptides are well studied in the evaluation of CHF and offer value independent of physician decision making [2-8], recent data have questioned the utility of routine natriuretic peptide determinations in severely dyspneic patients to improve clinical outcomes or diminish hospital resource utilization [9]. Additionally, the relationship between natriuretic peptides and the severity of heart failure appears less clear in patients with chronic kidney disease (CKD) and those with a history of prior CHF [10, 11].

The appropriate cut-off values for Amino Terminal pro Brain Natiuretic Peptide (NT-proBNP) for the diagnosis CHF were originally derived from the N-Terminal Pro BNP Investigation of Dyspnea in the Emergency Department (PRIDE) Study [7]. Broader standards were then put forth from pooled data in the International Collaborative of NT-proBNP (ICON) Study [8]. We undertook the present study to assess the diagnostic utility of NT-proBNP in a predominantly elderly, medically complex population presenting to the emergency room with dyspnea using accepted age-related cut points. We postulated that the utility of NT-proBNP in CHF would be diminished in elderly patients with multiple baseline cardiopulmonary co-morbidities.

## Methods

The study protocol was approved by our institutional review board. Data was reviewed for consecutive patients presenting to a university affiliated community hospital and have previously been presented at the 2006 American College of Cardiology Meeting [12]. We retrospectively studied 550 consecutive patients presenting to our emergency department in whom NT-proBNP values were obtained for the evaluation of dyspnea. Only subjects with incomplete laboratory results or missing clinical data were excluded from study. A total of 502 subjects were eligible for enrollment.

Data were collected and analyzed independently for each subject. Clinical characteristics including baseline demographics, presenting complaints, symptoms, signs, past medical history, medication use and examination findings were tabulated by a research assistant. Laboratory data and investigations including basic metabolic profile, complete blood count, electrocardiogram and chest x-ray were also recorded. Creatinine clearance was estimated using the Modification of Diet in Renal Disease method [13].

Determination of the diagnosis was made according to the Framingham Criteria for CHF [14] with the reviewing physician blinded to NT-proBNP determinations. The primary discharge diagnosis, secondary diagnosis and coded diagnosis from the patient visit was then reviewed to aid in the diagnosis of non-CHF events.

### Measurement of Levels of NT-proBNP

NT-proBNP analysis was conducted using established methods on a commercially available immunoassay (Elecsys proBNP, Roche Diagnostics, Indianapolis, Indiana).

### Statistical Analysis

Results were analyzed using standard statistical analysis including Mean (T-test), Kruskall-Wallis for medians, comparison of proportions for equality, and receiver operator curve (ROC) analysis. Commercially available SAS version 9.1 was used for analysis. Concentrations of proBNP were highly skewed and therefore logarithmically transformed before analysis. Resultant models were compared using log likelihood chi squared tests. Comparisons of NT-proBNP levels across diagnostic categories were performed using Kruskall-Wallis (non-parametric ANOVA) testing. The diagnostic performance of the assay was assessed by using receiver operating characteristic curves, formed by plotting sensitivity on the y axis and 1-specificity on the x axis for all possible cut off values of each diagnostic test.

The overall discriminatory ability of each test was shown by the area under the receiver operating characteristics curve. To test the accuracy in our cohort, the presence or absence of CHF was assessed based on the cut points derived from the previously pooled analysis [8]. We computed the sensitivity, specificity and diagnostic accuracy (defined as the sum of the concordant cells divided by the sum of all cells in two by two tables) of NT-proBNP measurements for all of the suggested cut-off points. Univariate and multivariate analyses were used to determine whether the peptide assay gave improved diagnosis of CHF over clinical predictors including age, gender, hematocrit and renal failure.

## Results

**Table 1:** Demographics and Clinical Characteristics in the Entire Cohort

	Total (%)	(-) CHF (%)	(+) CHF (%)	p-value
Demographics				
Age (years) (mean/range)	71 ± 15.1	70	73	0.05
Men (%)	45	46	45	0.78
Caucasian (%)	73	73	74	0.9
Symptoms/Signs				
Chest pain	22	23	22	0.8
Cough	17	20	15	0.16
Edema	9	6	11	0.03
Fever	7	7	7	0.88
Medical History				
Prior CHF	42	20	56	< 0.0001
Prior MI	19	11	24	< 0.001
Valvular Heart Disease	13	8	17	0.001
Coronary Artery Disease	37	29	42	< 0.01
Prior CABG/PCI	20	14	24	< 0.01
COPD/Prior PE	17	17	17	0.99
Systemic Hypertension	63	60	65	0.28
Diabetes Mellitus	36	29	40	0.01
Smoking History	18	19	17	0.44
Medications				
Spirinolactone	8	2	13	< 0.0001
Digoxin	17	10	22	< 0.001
Beta blocker	40	29	47	< 0.001
ACE-I/ARB	43	34	49	< 0.001
Loop diuretic	43	25	55	< 0.001
Other				
Hematocrit < 30	21	16	24	0.05
eGFR < 30	16	14	17	0.45

CHF: congestive heart failure; MI: myocardial infarction; PTCA: percutaneous coronary intervention; CABG: coronary artery bypass grafting; COPD: chronic obstructive pulmonary disease; PE: pulmonary embolus; ACE-I: angiotensin converting enzyme inhibitor; ARB: angiotensin receptor blocker; eGFR: estimated glomerular filtration rate. P value compares those with and without a final diagnosis of CHF.

The 502 consecutive patients meeting inclusion criteria comprised the study population. Forty eight subjects were excluded due to incomplete data collection. The demographics for the study are shown in Table 1. The mean age was 71.4 ± 15.1 years (range 20-104 years). Forty five percent (226) were male and 55% (276) were female. Prior CHF was present in 209 subjects (41.6%). Prior myocardial infarction was present in 18.5% and known coronary disease in 36.6% of patients. CHF was diagnosed in 301 (60%) of our subjects.

**Figure 1.**
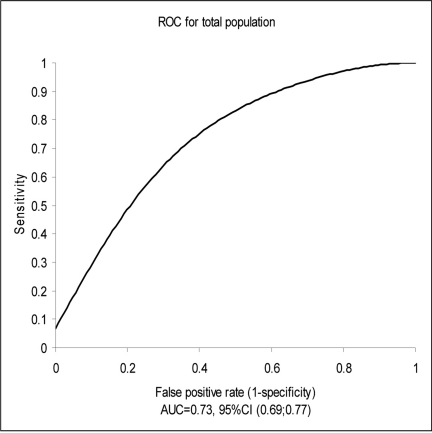
Test PerformanceArea Under the Receiver Operator Curve (ROC) Characteristics for Total Population. Median NT-proBNP for total cohort: 3050 pg/ml; Median NT-proBNP for CHF group: 4935 pg/ml; Median NT-proBNP for non-CHF group: 1075 pg/ml. CHF: Congestive Heart Failure.

The median NT-proBNP for the total cohort was 3050 pg/ml. Figure 1 demonstrates the receiver operator characteristics curve for the total population signifying test performance. The area under the curve (AUC) for NT-proBNP was 0.73 (95% CI: 0.65-0.75). Among patients who had a diagnosis of CHF, the median NT-proBNP level was 4935 pg/ml compared with 1075 pg/ml among patients without acute CHF (P < 0.001).

Table 2 shows the diagnostic analysis for the pooled international collaborative trial cut-pointfs in our cohort for the diagnosis of CHF. The cumulative data demonstrated a sensitivity of 81% and a specificity of 53% in our population, with a positive predictive value of 72% and a diagnostic accuracy of 70%. The diagnostic accuracy of NT-proBNP in our study appears somewhat inferior to the collaborative groups findings (70% vs. 83%, respectively). Additionally, the negative predictive value of a single age-independent cut point of 300 pg/ml was also less (86%) in our study as compared to 98% in the ICON study.

**Table 2:** Analysis Based on the ICON Trial Cut-offs

Cutoff Value Age (years) NT-proBNP	Sensitivity (%)	Specificity (%)	Positive Predictive Value (%)	Negative Predictive Value (%)	Diagnostic Accuracy (%)
<50 years 450 pg/ml	94	45	72	82	65
50-75 years 900 pg/ml	80	56	69	70	67
>75 years 1800 pg/ml	79	52	75	59	70
Cumulative Data	81	53	72	66	70

ICON: International Collaborative of NT-proBNP

**Figure 2.**
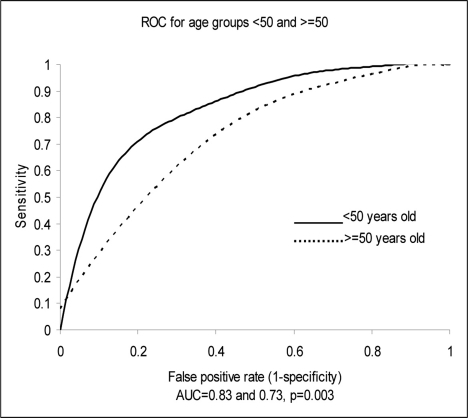
Test performance-area under the receiver operator curve (ROC) characteristics for age 50 or ≥ 50 years.

**Figure 3.**
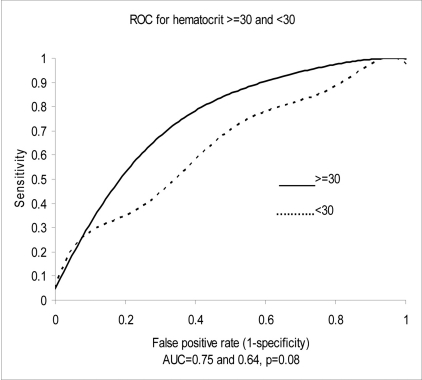
Test performance-area under the receiver operator curve (ROC) characteristics for hematocrit.

Univariate analysis was used to assess the diagnostic accuracy of NT-proBNP for CHF according to age, the presence of renal disease or anemia. Age (> 50 years) and renal insufficiency (creatinine clearance of < 30 ml/min) correlated directly with NT-proBNP levels, while anemia (hematocrit < 30%) correlated inversely with NT-proBNP levels. Creatinine clearance estimated of < 30 ml/min did not, however, significantly affect the AUC or diagnostic accuracy of NT-proBNP, while age (> 50 years) shifted the AUC from 0.83 to 0.73 (P ≤ 0.01). Anemia (hematocrit < 30%) had a borderline effect on the diagnostic accuracy of NT-proBNP, shifting the AUC from 0.75 to 0.64 (P = 0.08) (Fig. 2, 3).

By multivariate linear regression both age and creatinine clearance were highly significant predictors of NT-proBNP levels (P < 0.0001), while hematocrit was of borderline significance (P = 0.06). In a multivariate model including NT-proBNP, age, prior CHF, anemia and renal insufficiency the best predictor of CHF was prior CHF, odds ratio (OR) = 45; 95% CI: 23-88. The OR for NT-proBNP remained significant (OR = 5; 95% CI: 2.5-10), while the OR for anemia was of borderline significance (OR = 1.7; 95% CI: 0.9-3.2).

## Discussion

Our findings demonstrate that NT-proBNP determinations, although useful for the diagnosis of CHF in a complex elderly population with multiple comorbidities, may be less discriminating than previously thought. Although the diagnostic accuracy of age-adjusted NT-proBNP cut off values appears less robust in this population, the negative predictive value of a single age-independent value of 300 pg/ml remained reasonably high at 86%. In multivariate analysis, prior CHF was the most potent predictor of CHF in an elderly emergency room population presenting with dyspnea. Utilizing age-adjusted algorithms derived from the Pride study, the ICON study reported a sensitivity of 90% and a specificity of 84% for NT-proBNP, with a negative predictive value of 66%, a positive predictive value of 88% and diagnostic accuracy of 83% for CHF in a large pooled database [8]. Overall the collaborative results appear superior to our findings.

Compared to the ICON study, our cohort involved an older population (mean age 71 vs. 68 years) with a higher incidence of cardiovascular co-morbidities, especially prior CHF (42% vs. 34%, respectively). The difference in the prevalence of prior CHF in our cohort as compared to the original PRIDE study (42% vs. 25% respectively) was even more striking. It is of particular interest that the accuracy of NT-proBNP in our study was nearly identical to that of Chung et als [10] study of patients with prior CHF (AUC 0.73 vs. 0.75, respectively).

As advancing age is associated with reduced ventricular compliance [15] and glomerular filtration rates [16], it is not surprising that age has been previously shown to be directly correlated with both BNP and NT-proBNP levels. Recent data has shown a close correlation between estimated GFR and NT-proBNP levels [17]. Both BNP and NT-proBNP are equally dependent on renal function for their clearance [18] and CKD may be associated with cardiac pathology of important clinical significance. Additionally, the prevalence of primary pulmonary causes of dyspnea was relatively low in our cohort (< 10%) as compared to the ICON study (37%). Finally, a final diagnosis of CHF was made in 35% of PRIDE subjects versus 60% of our subjects revealing further differences in these patient populations.

Several other recent studies have also suggested that higher cut-offs should be used for elderly as compared to younger patients to improve specificity [19, 20]. Similarly, the diagnostic accuracy of NT-proBNP in these studies was significantly better than what was seen in our population. Utilizing cutoffs of 972 pg/ml and 2000 pg/ml, respectively for younger as compared to elderly patients in a population with a mean age of approximately 70 years, these authors obtained diagnostic accuracies of 91% and 83%, which are similar to the PRIDE and pooled data.

Although NT-proBNP performed similarly to BNP in the general population in detecting left ventricular dysfunction [21], a recent comparison of BNP and NT-proBNP in the diagnosis of CHF in patients age 65 and older [22] suggested that NT-proBNP may be less useful in this population. Although there was a strong correlation between both natriuretic peptides in the general population, BNP determinations were more accurate in the diagnosis of CHF. Similarly, concordance between the 2 natriuretic peptides was found in blood samples taken from an emergency room population, but renal impairment affected only the NT-proBNP results [23].

In our cohort although renal function correlated directly with NT-proBNP levels, reduced creatinine clearance did not affect the diagnostic accuracy of proBNP testing. Similar findings were noted in a subgroup analysis in PRIDE [24] where the area under the ROC remained unchanged in patients with moderate renal insufficiency compared to those with severe renal insufficiency. Similar data exists on the effect of creatinine clearance on the diagnostic accuracy of CHF for BNP [25-27]. Several studies have shown an independent effect of renal function on BNP in the absence of elevated left ventricular filling pressures [25, 26], while McCullough et al. reported that renal function influences the cut point for BNP in the diagnosis of CHF [27].

Hematocrit < 30% may also adversely affect the diagnostic accuracy NT-proBNP. Our data are consistent with the previously described inverse relationship of hematocrit to absolute NT-proBNP levels [28, 29]. A change in the area under the receiver operating curve from 0.75 to 0.64 (P = 0.08) with a HCT < 30% demonstrates a potential confounding factor affecting test accuracy. Willis et al. [30] showed that anemia was associated with elevated NT-proBNP above cut-off levels in adults without CHF or renal disease, while Muscari et al. [31] showed that the relationship between hematocrit and NT-proBNP was independent of echocardiographic variables. Similarly, hemoglobin levels are independent predictors of BNP levels in the absence of CHF [32].

NT-proBNP is a useful adjunct to clinical and radiographic assessment of heart failure and provides prognostic information regarding re-hospitalization risk and mortality [33]. However, it is important to remember that the diagnostic accuracy of any test should be validated for the specific population being studied. Individual hospitals should assess the diagnostic accuracy of natriuretic peptide assays in their specific patient populations to better understand the strengths and limitations of their use. Older subjects with an increased prevalence of prior CHF and cardiovascular disease, as well as anemia and renal disease, may have chronically elevated NT-proBNP levels which diminishes the positive predictive value for NT-proBNP in that population. Utilizing higher age-adjusted cut-off values for NT-proBNP may improve the positive predictive value of NT-proBNP, but may adversely affect the negative predictive value and overall diagnostic accuracy.

### Limitations

Our study results are limited by the retrospective design of the trial and modest sample size from a single center. False negative NT-proBNP levels may also occur in obesity, but body mass index calculations were not available for analysis in our study. Our results may have also been biased by patient selection with a high prevalence of prior CHF patients. We analyzed only subjects in whom a NT-proBNP sample was drawn and NT-proBNP utilization in subjects with primary pulmonary pathology appears lower in our study as compared to prior reports. Due to the high prevalence of cardiac morbidities and low prevalence of pulmonary morbidities, generalization of our results may be limited. Patients were not followed prospectively after hospital discharge and thus the prognostic value of NT-proBNP could not be ascertained in our population.

### Conclusion

The diagnostic accuracy of NT-proBNP in the evaluation of dyspnea utilizing current age-specific cut-points appears to be diminished as compared to prior studies in elderly subjects with a high prevalence of prior CHF and cardiovascular comorbidities. Age, prior history of CHF and hematocrit levels appear to be the most important determinants of NT-proBNP diagnostic accuracy and deserve further study. Although renal insufficiency is associated with elevated NT-proBNP determinations, creatinine levels do not appear to materially affect the diagnostic accuracy of the assay. Further studies are needed in larger samples of patients to assess the diagnostic accuracy of NT-proBNP in elderly subjects and the effect of prior cardiovascular disease, anemia, and renal function on assay performance.

## References

[1] Silver MA, Maisel A, Yancy CW, McCullough PA, Burnett JC, Francis GS, Mehra MR (2004). BNP Consensus Panel 2004: A clinical approach for the diagnostic, prognostic, screening, treatment monitoring, and therapeutic roles of natriuretic peptides in cardiovascular diseases. Congest Heart Fail.

[2] McCullough PA, Nowak RM, McCord J, Hollander JE, Herrmann HC, Steg PG, Duc P (2002). B-type natriuretic peptide and clinical judgment in emergency diagnosis of heart failure: analysis from Breathing Not Properly (BNP) Multinational Study. Circulation.

[3] Baggish AL, Cameron R, Anwaruddin S, Chen AA, Krauser DG, Tung R, Januzzi JL (2004). A clinical and biochemical critical pathway for the evaluation of patients with suspected acute congestive heart failure: The ProBNP Investigation of Dyspnea in the Emergency Department (PRIDE) algorithm. Crit Pathw Cardiol.

[4] Hunt SA, Abraham WT, Chin MH, Feldman AM, Francis GS, Ganiats TG, Jessup M (2009). 2009 Focused update incorporated into the ACC/AHA 2005 Guidelines for the Diagnosis and Management of Heart Failure in Adults A Report of the American College of Cardiology Foundation/American Heart Association Task Force on Practice Guidelines Developed in Collaboration With the International Society for Heart and Lung Transplantation. J Am Coll Cardiol.

[5] Maisel AS, Krishnaswamy P, Nowak RM, McCord J, Hollander JE, Duc P, Omland T (2002). Rapid measurement of B-type natriuretic peptide in the emergency diagnosis of heart failure. N Engl J Med.

[6] Maisel A, Hollander JE, Guss D, McCullough P, Nowak R, Green G, Saltzberg M (2004). Primary results of the Rapid Emergency Department Heart Failure Outpatient Trial (REDHOT). A multicenter study of B-type natriuretic peptide levels, emergency department decision making, and outcomes in patients presenting with shortness of breath. J Am Coll Cardiol.

[7] Januzzi JL, Camargo CA, Anwaruddin S, Baggish AL, Chen AA, Krauser DG, Tung R (2005). The N-terminal Pro-BNP investigation of dyspnea in the emergency department (PRIDE) study. Am J Cardiol.

[8] Januzzi JL, van Kimmenade R, Lainchbury J, Bayes-Genis A, Ordonez-Llanos J, Santalo-Bel M, Pinto YM (2006). NT-proBNP testing for diagnosis and short-term prognosis in acute destabilized heart failure: an international pooled analysis of 1256 patients: the International Collaborative of NT-proBNP Study. Eur Heart J.

[9] Schneider HG, Lam L, Lokuge A, Krum H, Naughton MT, De Villiers Smit P, Bystrzycki A (2009). B-type natriuretic peptide testing, clinical outcomes, and health services use in emergency department patients with dyspnea: a randomized trial. Ann Intern Med.

[10] Chung T, Sindone A, Foo F, Dwyer A, Paoloni R, Janu MR, Wong H (2006). Influence of history of heart failure on diagnostic performance and utility of B-type natriuretic peptide testing for acute dyspnea in the emergency department. Am Heart J.

[11] Vanderheyden M, Bartunek J, Filippatos G, Goethals M, Vlem BV, Maisel A (2008). Cardiovascular disease in patients with chronic renal impairment: role of natriuretic peptides. Congest Heart Fail.

[12] Afaq MA, Shoraki A, Ivanov O, Srinivasan J, Mayall I, Bernstein L, Choi Y, Zarich S (2006). Diagnostic utility of NT-proBNP in the evaluation of congestive heart failure in the emergency department in elderly patients with multiple co-morbidities. J Amer Coll Cardiol.

[13] Levey AS, Coresh J, Greene T, Marsh J, Stevens LA, Kusek JW, Van Lente F (2007). Expressing the Modification of Diet in Renal Disease Study equation for estimating glomerular filtration rate with standardized serum creatinine values. Clin Chem.

[14] McKee PA, Castelli WP, McNamara PM, Kannel WB (1971). The natural history of congestive heart failure: the Framingham study. N Engl J Med.

[15] Arbab-Zadeh A, Dijk E, Prasad A, Fu Q, Torres P, Zhang R, Thomas JD (2004). Effect of aging and physical activity on left ventricular compliance. Circulation.

[16] Kistorp C, Raymond I, Pedersen F, Gustafsson F, Faber J, Hildebrandt P (2005). N-terminal pro-brain natriuretic peptide, C-reactive protein, and urinary albumin levels as predictors of mortality and cardiovascular events in older adults. JAMA.

[17] Bernstein LH, Zions MY, Haq SA, Zarich S, Rucinski J, Seamonds B, Berger S (2009). Effect of renal function loss on NT-proBNP level variations. Clin Biochem.

[18] van Kimmenade RR, Januzzi JL, Bakker JA, Houben AJ, Rennenberg R, Kroon AA, Crijns HJ (2009). Renal clearance of B-type natriuretic peptide and amino terminal pro-B-type natriuretic peptide a mechanistic study in hypertensive subjects. J Am Coll Cardiol.

[19] Bayes-Genis A, Santalo-Bel M, Zapico-Muniz E, Lopez L, Cotes C, Bellido J, Leta R (2004). N-terminal probrain natriuretic peptide (NT-proBNP) in the emergency diagnosis and in-hospital monitoring of patients with dyspnoea and ventricular dysfunction. Eur J Heart Fail.

[20] Lainchbury JG, Campbell E, Frampton CM, Yandle TG, Nicholls MG, Richards AM (2003). Brain natriuretic peptide and n-terminal brain natriuretic peptide in the diagnosis of heart failure in patients with acute shortness of breath. J Am Coll Cardiol.

[21] Costello-Boerrigter LC, Boerrigter G, Redfield MM, Rodeheffer RJ, Urban LH, Mahoney DW, Jacobsen SJ (2006). Amino-terminal pro-B-type natriuretic peptide and B-type natriuretic peptide in the general community: determinants and detection of left ventricular dysfunction. J Am Coll Cardiol.

[22] Ray P, Arthaud M, Birolleau S, Isnard R, Lefort Y, Boddaert J, Riou B (2005). Comparison of brain natriuretic peptide and probrain natriuretic peptide in the diagnosis of cardiogenic pulmonary edema in patients aged 65 and older. J Am Geriatr Soc.

[23] Sykes E, Karcher RE, Eisenstadt J, Tushman DA, Balasubramaniam M, Gusway J, Perason VJ (2005). Analytical relationships among Biosite, Bayer, and Roche methods for BNP and NT-proBNP. Am J Clin Pathol.

[24] Anwaruddin S, Lloyd-Jones DM, Baggish A, Chen A, Krauser D, Tung R, Chae C (2006). Renal function, congestive heart failure, and amino-terminal pro-brain natriuretic peptide measurement: results from the ProBNP Investigation of Dyspnea in the Emergency Department (PRIDE) Study. J Am Coll Cardiol.

[25] Forfia PR, Watkins SP, Rame JE, Stewart KJ, Shapiro EP (2005). Relationship between B-type natriuretic peptides and pulmonary capillary wedge pressure in the intensive care unit. J Am Coll Cardiol.

[26] Tsutamoto T, Wada A, Sakai H, Ishikawa C, Tanaka T, Hayashi M, Fujii M (2006). Relationship between renal function and plasma brain natriuretic peptide in patients with heart failure. J Am Coll Cardiol.

[27] McCullough PA, Duc P, Omland T, McCord J, Nowak RM, Hollander JE, Herrmann HC (2003). B-type natriuretic peptide and renal function in the diagnosis of heart failure: an analysis from the Breathing Not Properly Multinational Study. Am J Kidney Dis.

[28] Willis MS, Lee ES, Grenache DG (2005). Effect of anemia on plasma concentrations of NT-proBNP. Clin Chim Acta.

[29] Muscari A, Berzigotti A, Bianchi G, Giannoni C, Ligabue A, Magalotti D, Sbano D (2006). Non-cardiac determinants of NT-proBNP levels in the elderly: relevance of haematocrit and hepatic steatosis. Eur J Heart Fail.

[30] Wold Knudsen C, Vik-Mo H, Omland T (2005). Blood haemoglobin is an independent predictor of B-type natriuretic peptide (BNP). Clin Sci (Lond).

[31] Baggish AL, van Kimmenade R, Bayes-Genis A, Davis M, Lainchbury JG, Frampton C, Pinto Y (2007). Hemoglobin and N-terminal pro-brain natriuretic peptide: Independent and synergistic predictors of mortality in patients with acute heart failure Results from the International Collaborative of NT-proBNP (ICON) Study. Clin Chim Acta.

[32] Vickery S, Price CP, John RI, Abbas NA, Webb MC, Kempson ME, Lamb EJ (2005). B-type natriuretic peptide (BNP) and amino-terminal proBNP in patients with CKD: relationship to renal function and left ventricular hypertrophy. Am J Kidney Dis.

[33] Daniels LB, Maisel AS (2007). Natriuretic peptides. J Am Coll Cardiol.

